# Haloarchaea as Promising Chassis to Green Chemistry

**DOI:** 10.3390/microorganisms12081738

**Published:** 2024-08-22

**Authors:** Emma Bonnaud, Philippe M. Oger, Avigaël Ohayon, Yoann Louis

**Affiliations:** 1 SEGULA Technologies, 13 Bis Avenue Albert Einstein, 69100 Villeurbanne, France; emma.bonnaud@insa-lyon.fr (E.B.);; 2 INSA de Lyon, UMR5240 CNRS, Université Claude Bernard Lyon 1, 11, Avenue Jean Capelle, 69621 Villeurbanne, France

**Keywords:** haloarchaea, cellular chassis, green chemistry, extremozymes, genetic tools, genetic modifications

## Abstract

Climate change and the scarcity of primary resources are driving the development of new, more renewable and environmentally friendly industrial processes. As part of this green chemistry approach, extremozymes (extreme microbial enzymes) can be used to replace all or part of the chemical synthesis stages of traditional industrial processes. At present, the production of these enzymes is limited by the cellular chassis available. The production of a large number of extremozymes requires extremophilic cellular chassis, which are not available. This is particularly true of halophilic extremozymes. The aim of this review is to present the current potential and challenges associated with the development of a haloarchaea-based cellular chassis. By overcoming the major obstacle of the limited number of genetic tools, it will be possible to propose a robust cellular chassis for the production of functional halophilic enzymes that can participate in the industrial transition of many sectors.

## 1. Introduction

Climate change, societal pressure and the scarcity of primary resources are driving manufacturers to develop new, more renewable and environmentally friendly industrial processes. It is with this in mind that green chemistry has emerged. Green chemistry aims to reduce or eliminate the use and synthesis of hazardous substances throughout the lifecycle of a chemical product. Enzymes fit perfectly into this approach, offering a renewable alternative to chemical catalysis. Their energy requirements are lower, the resulting production routes are reduced, and their use limits the production of waste. In addition, their functional and operational diversity makes it possible to replace most chemical synthesis steps with enzymatic equivalents [1]. In recent years, the demand for enzymes has therefore increased, with the global market quadrupling in 15 years and expected to reach USD 14.5 billion by 2027 [2].

However, their use as industrial biocatalysts is currently limited by the extreme conditions found in industry. Industrial enzymes are mostly mesophilic and therefore perform poorly in extreme conditions. It is therefore essential to focus on the deployment of extremozymes in industry. These enzymes, isolated from extremophilic organisms, have structural adaptations that allow them to be active and functional under industrial conditions. Today, the lack of efficient expression systems is an obstacle to exploiting the potential of these enzymes. In fact, their production is limited to conventional methods, based on mesophilic bacterial hosts such as *Bacillus subtilis*. However, the culture conditions and transcription/translation mechanisms of these hosts are not always compatible with the overproduction of extremozymes. This is particularly true for halophilic enzymes (enzymes isolated from organisms adapted to high salinity), which are used in the detergent, textile and food industries and cannot be produced in their active forms under the low-salt conditions required for the growth of most protein overexpression chassis [3]. Indeed, the lack of host resistance to high salinity leads to extensive purification treatments that are incompatible with industry [4,5]. It is therefore crucial to develop new cellular chassis, based on other types of organisms, whose growth and genetic and post-translational modification abilities are compatible with the overproduction of these extremozymes. These chassis can be based on different species of extremophilic archaea, which enables the overproduction of extremozymes under targeted industrial conditions. In the specific case of haloenzymes, their production requires the construction of a salt-adapted cellular chassis.

The objective of this review is to discuss the potential use of haloarchaea as a cellular chassis. It will give an overview of the genetic tools developed in salt-loving archaea and those still to be developed to propose a robust chassis to apply in industry.

## 2. Presentation of Haloarchaea

Halophiles, extremophile microorganisms, are found in all three domains of life (Eukarya, Bacteria and Archaea), but archaea represent the vast majority of them [6,7]. Haloarchaea are mostly obligate halophiles, facultative aerobes, heterotrophs, prototrophic and slightly thermophilic [6]. They grow in hypersaline ecosystems (environments with a higher salt concentration than sea water), with optimal growth in conditions with 10–35% (1.71 to 6 M) NaCl [7]. Haloarchaea have been isolated from saline environments with a wide pH range, ranging from neutral (such as Lake Afrera in Ethiopia, pH of 6.55) [8] to very high-pH environments (pH > 9, such as Lake Magadi in Africa) [6]. Among the haloarchaea, the order *Halobacteriales*, which includes the genera *Halobacterium*, *Halococcus*, *Haloarcula*, *Haloferax* and *Natronococcus*, can be cited as an example.

### 2.1. Osmotic Adaptation

To cope with the osmotic potential of their habitat, these archaea adopt one of two strategies: the accumulation of a molar concentration of K^+^ and Cl^−^ (salt-in strategy), or the accumulation of an organic osmotic solute (salt-out strategy) [9].

Whatever the strategy, sodium ions are expelled from the cytoplasm (as much as possible because these ions are detrimental to the functioning of halophilic cells), and this is usually performed with the help of Na^+^/H^+^ antiporters.

**Figure 1:**
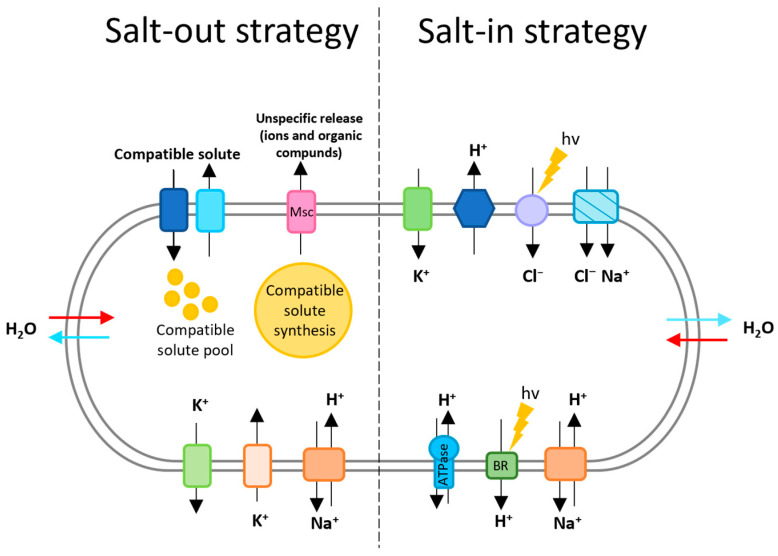
Schematic illustration of the salt-out strategy adopted by some haloarchaea. Representation of the mechanisms of adaptation to high salinities. To cope with the high potential of these environments, two strategies can be adopted by halophilic archaea. The salt-out strategy is divided into two parts. It starts with an immediate adjustment response, consisting of the cellular import of K^+^ (acute osmotic stress). The second phase starts with the import or de novo synthesis of the compatible solute [11]. During this phase, Na^+^ and K^+^ are excluded from the cytoplasm. Cells also have efflux systems for compatible solutes (turquoise transporter, which may adjust the turgor during allotment and doubling before cell division) and Msc channels (mechanosensitive channels, which serve as safety valves, allowing the rapid release of ions and organic solutes in the case of sudden downward osmotic shocks) [13]. The salt-in strategy is implemented by sequestering cations in the cytoplasm [12]. For this, Cl^−^ is transported into the cytoplasm with the help of primary or secondary transporters (halorhodopsin, light-driven chloride pump in purple and symporter in blue), and potassium is absorbed with K^+^ uniport (green transporter), driven by membrane potential [14]. Whatever the strategy, sodium ions are expelled from the cytoplasm by Na^+^/H^+^ antiporters [9]. The most important transporters are summarized in this figure; they include ion pumps (e.g., Cl^−^ pump in purple), uniport (K^+^ uniport), antiporters (such as Na^+^/H^+^ antiporter), bacteriorhodopsin (membrane bound proton pump), symporter (chloride transport probably coupled with inward transport of sodium), ATP synthase (ATP formation) and respiratory chain (proton translocation) [9]. The red and blue arrows represent the fluxes of water in ad out of the cell respectively.

The salt-out strategy consists of the exclusion of sodium from the cytoplasm and the accumulation of a high concentration of organic solutes (sugars, sugar derivates, polyalcohols, …), amino acid derivates or compatible solutes (ectoin and derivates) (Figure 1). These compounds are absorbed from the environment (for organic solutes) or synthesized de novo to increase the internal osmolarity without increasing the cytoplasmic salinity [10,11]. The mode of action of these solutes is not fully elucidated. They could act by maintaining the osmolarity of the cell with respect to its environment or they could also protect the proteins (by allowing them a cytoplasmic localization without particular adaptation). Due to its high energy cost for the cell, it is less adapted to saturating salt concentrations [12].

For the salt-in strategy, an electrochemical gradient of protons is set up. It is, in particular, set up by the respiratory chain that, by the transport of electrons (with the electron acceptor), involves the extrusion of protons (salt-in strategy, Figure 1). The haloarchaea presenting the bacteriorhodopsin (protein retinene localized at the level of the membrane) are also able to generate this gradient with the help of the light (Figure 1). The establishment of this gradient allows the formation of Adenosine triphosphate (ATP) by the membrane ATP synthase. The latter couples the phosphorylation of Adenosine diphosphate (ADP) to an incoming flow of H^+^. In the case of anaerobic growth, the proton gradient is set up by ATP synthase, which works in the opposite direction [9].

Potassium, the predominantly accumulated ion in this strategy, enters the cell through a uniport system (in response to membrane potential) (Figure 1). Potassium is taken up upon the expulsion of Na^+^ (maintenance of electroneutrality). As described above, sodium is excluded from the cytoplasm with the help of an Na^+^/H^+^ antiporter that uses the electrochemical proton gradient as a driving force (Figure 1) [9].

Finally, Cl^−^ uptake occurs with the help of two energy-dependent pumps. These are a Cl^−^/Na^+^ symport (light-independent transport) and a primary light-dependent Cl^−^ pump (this is the retinal protein halorhodopsin) (Figure 1) [9].

For more details on this strategy, see Oren, 2006. This strategy requires a number of physiological changes to maintain the full regulatory and metabolic functions of the cell (adaptation of enzymes and cellular components to high salinity). Typically, microorganisms employing this strategy (obligate halophile of which the archaea are a part) exhibit an acidified proteome and high GC content. The acidification of the proteome would be essential for protein solubility under such environmental conditions [10].

Although extreme halophiles prefer the salt-in strategy, recent works show the use of the salt-out strategy (used by halotolerant organisms) in certain situations [15,16]. As an example, Youssef et al., contradicted the previously general idea that all haloarchaea would adopt only the salt-in strategy and demonstrated that the salt-out strategy is a common mechanism of osmoadaptation in the order *Halobacteriales* [16]. The study of Youssef et al., shows that the production of trehalose or 2-sulfothrealose and the absorption of glycine betaine is widespread in *Halobacteriales*. Genes encoding the trehalose biosynthetic pathway and genes encoding the glycine-betaine Betaine/Carnitine/Choline Transporter (BCCT) family transporters were found in 38 and 60 *Halobacteriales* genomes, respectively. Nevertheless, it appears that permanent hypersaline environments harbor genus lacking trehalose production [16]. The need to synthesize and accumulate increasing amounts of organic solutes at higher salinities makes this strategy energetically unfavorable. This is why haloarchaea favors the salt-in strategy at high salt concentrations in the medium [12].

Proteins are able to function in hypersaline environments by reducing the overall hydrophobicity and increasing surface acid residues. The high number of negative charges on the surface coordinates a network of hydrated cations and keeps the protein in solution. Due to their high content of negatively charged amino acids, halophilic proteins can unfold in the absence of an optimal salt concentration [4,7].

### 2.2. Haloarchaea: Invaluable Resources of Enzymes for Industry

Haloarchaeal enzymes are an invaluable resource for industry due to their polyextremophily. In addition to their resistance to high salinity, they are very often resistant to extreme pH levels, high temperatures, low water activity, etc. [7,17].

Environments are considered hypersaline when the salt concentration exceeds that of seawater (i.e., a concentration greater than 35 g/L), and they have a characteristic ionic composition (anions such as chlorine and bromide, and cations such as calcium and potassium) and organic molecules such as acetate [18]. These environments are very common in industry, particularly in the detergent, textile and other industries. Haloarchaeal enzymes are particularly well adapted to these environments. Due to their osmoadaptation strategy, haloarchaea, unlike halobacteria, produce intracellular enzymes adapted to high salinity, while the later produce only extracellular salt-adapted enzymes. For example, lipases and esterases from *Haloarcula marismortui* and *Natronococcus sp*. TC6 are widely used in the biofuels, detergents and textiles sectors [7]. Other examples include glutamate dehydrogenase from *Halobacterium salinarum*, which operates optimally at a temperature of 70 °C, a pH of 8.5–9.2 and a NaCl concentration of 3–3.5 M, or alcohol dehydrogenase from *Natronomonas pharaonis*, which operates optimally at a temperature of 70 °C, a pH of 8–10 and a NaCl concentration of 5 M [5]. Although not yet used in industry, these enzymes will be excellent biocatalysts due to their polyextremophilic properties.

Organic solvents, commonly used in industry, tend to reduce water activity. These solvents are used in many sectors, including the chemical and paint industries. Haloarchaea are known to be resistant to low water activity (high salinity can reduce water activity by 1 to 0.75), thus extending their range of applications [7].

Industry consumes significant amounts of freshwater (about 20%) [19]. However, unlike seawater, freshwater reserves are not inexhaustible. It would then be necessary to substitute freshwater for seawater in industry to reduce the water footprint of this sector. Although the salt concentration in seawater is well below the optimum concentration for haloarchaea, some haloarchaea have been isolated from seawater (e.g., *Haloferax marinum*) [20]. It would then be possible to replace fresh water with seawater by using halophilic enzymes, making bioprocesses more sustainable and conserving freshwater for vital uses [21]. Limiting the use of freshwater reduces the costs of production, which is a very important if we want a bioprocess to be viable. This approach would then be in line with the measures taken by various countries to combat water stress.

Haloarchaeal enzymes are therefore excellent biocatalysts and could replace a wide range of chemical catalysts. However, the production of these enzymes is still the main bottleneck. As these enzymes are functional at high salinities, it is necessary to use a host with a salt-in osmoadaptation strategy. The use of hosts that adopt a salt-out strategy, as most of the current protein overproduction chassis do, leads to the formation of inclusion bodies and soluble inactive proteins, and therefore requires additional purification steps [5,22]. It is therefore necessary to develop a haloarchaeal chassis (implementing a salt-in strategy) to enable the efficient production of halophilic enzymes [7].

## 3. Haloarchaea Chassis: Current State and Challenges

### 3.1. Specifications for a Haloarchaea Chassis

**Figure 2:**
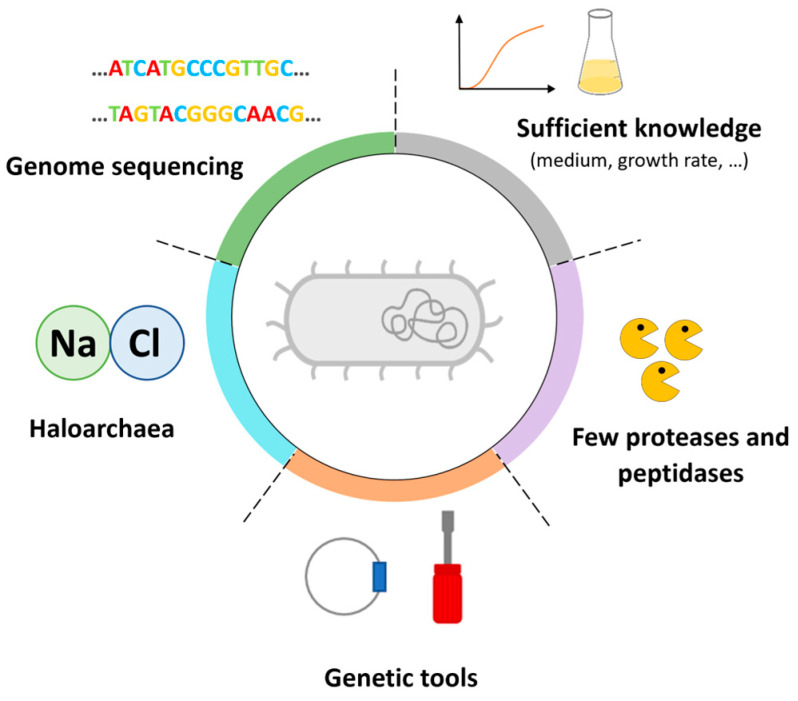
Haloarchaea chassis specification.

A haloarchaeal chassis must fulfil several conditions to be considered an ideal chassis (Figure 2). First, (i) sufficient knowledge of the microorganism of interest is required (such as the culture medium, generation time, etc.) [23]. (ii) A wide range of genetic manipulation tools (transformation methods, plasmid vectors, promoter libraries, etc.) are required to achieve the optimal production of the enzymes [23,24]. This makes it possible to modify the host organism, if necessary, in order to deplete degradation systems (the deletion of proteases capable of degrading the proteins of interest and restriction/modification systems so as not to interfere with the supply of exogenous DNA), to improve the folding of the proteins of interest (molecular chaperones, codon bias, etc) and to optimize transport systems (e.g., it is possible to fuse a signal peptide to the protein encoding our protein of interest) [4,23,24]. This also requires (iii) the genome of the host organism to be completely sequenced [23,24]. Finally, (iv) to facilitate construction, the host must provide native plasmids (basis for vector construction) and (v) a limited number of proteases/peptidases (to limit the degradation of the overproduced enzyme).

### 3.2. The Number of Potential Haloarchaea Chassis Is Limited by the Genetic Tools Available

To highlight the potential salt-loving chassis, Table S1 [25,26,27,28,29,30,31,32,33,34,35,36,37,38,39,40,41,42,43,44,45,46,47,48,49,50,51,52,53,54,55,56,57,58,59,60,61,62,63,64,65,66,67,68,69,70,71,72,73,74,75,76,77,78,79,80,81,82,83,84,85,86,87,88,89] summarizes the haloarchaeal species for which information on the genome, genetic tools and culture conditions was found. Among these species, only a few have been studied for the development of genetic tools, thus limiting the potential chassis (Table S1) [25,26,27,28,29,30,31,32,33,34,35,36,37,38,39,40,41,42,43,44,45,46,47,48,49,50,51,52,53,54,55,56,57,58,59,60,61,62,63,64,65,66,67,68,69,70,71,72,73,74,75,76,77,78,79,80,81,82,83,84,85,86,87,88,89]. The existence of genetic tools is an important aspect for the development of a cellular chassis. Furthermore, although genetic tools are available for 12 haloarchaeal species (Table S1) [25,26,27,28,29,30,31,32,33,34,35,36,37,38,39,40,41,42,43,44,45,46,47,48,49,50,51,52,53,54,55,56,57,58,59,60,61,62,63,64,65,66,67,68,69,70,71,72,73,74,75,76,77,78,79,80,81,82,83,84,85,86,87,88,89], only two species currently have enough tools to imagine the development of a cellular chassis. *Haloferax volcanii* and *Hbt. salinarum* are the only species for which inducible promoters or a wide range of vectors are available. However, despite the relatively large number of tools available in *Hbt. salinarum*, this strain has slow growth, a poorly developed set of selectable markers, and an unstable genome (frequent IS-mediated rearrangements) [12]. For these reasons, it is ill-suited as a cellular chassis for protein overproduction in an industrial context. In contrast, because of its rapid growth, ease of handling and existing genetic tools, *Hfx. volcanii* is a good candidate for the construction of a cellular chassis. However, additional genetic modifications and the development of genetic tools that are more suitable for industrial production would be required before it could be considered as a cellular chassis. It is also important to extend the genetic tools to other haloarchaeal species. Many haloarchaea are polyextremophilic (e.g., haloalkaliphilic archaea). By targeting specific species, it will be possible to propose cellular chassis capable of producing enzymes that are resistant to multiple conditions, thus reaching more industrial sectors.

### 3.3. Haloarchaea Chassis Construction: Genetic Tools Available

There is currently no haloarchaeal cellular chassis available. The development of such a chassis requires a wide range of genetic tools. Firstly, it requires the genetical modification of the host to make it compatible with the overproduction of the product of interest, and secondly, it requires the construction of an overproduction vector. The genetic tools available for haloarchaea are very limited. This is due to the difficulty of adapting existing genetic tools to archaea. These are often resistant to conventional antibiotics used in bacteria because the targets of these antimicrobials are often absent in archaea. In addition, the extreme salinity under which haloarchaea develop generally means that traditional reporting systems are ill-adapted, because these mesophilic reporting systems fail to fold properly and function under high-salt conditions.

#### 3.3.1. Transformation Methods

The transformation of haloarchaea is based on the formation of spheroplasts and the use of polyethylene glycol 600 (PEG_600_). Spheroplasts are formed by removing the glycoprotein layer from the cell surface (S-layer) with ethylene diamine tetra-acetic acid (EDTA) [31,67,79,90]. This method is slightly different for haloalkaliphilic archaea (salt and alkalinity resistance), where spheroplasts are formed by the action of bacitracin, proteolytic enzymes and EDTA [67,68,69,70,71,72,73,74,75,76,77,78,79]. Once the spheroplasts are formed, DNA is introduced using PEG_600_, which disrupts the selective permeability of the plasma membrane. The cells are then transferred to a rich broth before being placed on selective medium. The efficacy of this protocol has been demonstrated in numerous studies [4,45,67,79,90].

#### 3.3.2. Selectable Markers

Selectable markers are generally genes that confer resistance to an antibiotic or genes that ensure the prototrophy of auxotrophic strains in environments lacking essential nutrients [32]. Only a few antibiotics are capable of interfering with the growth of haloarchaea. The identification of antibiotic resistance genes has led to the development of two selectable markers for these archaea. These are novobiocin, which inhibits the β-subunit of DNA gyrase, and mevinolin (or simvastatin), which targets 3-hydroxy-3-methylglutarym coenzyme A reductase (HMG-CoA). These antibiotics inhibit functions essential for DNA and archaeal membrane synthesis [32]. The main disadvantage of these selectable markers is the risk of spontaneous mutations. These mutations can occur as a result of point mutations in the gene promoter or by amplification of the chromosomal gene [12,32]. In addition, recombination between the resistance gene and the chromosome can occur. This is particularly the case for the mevinolin resistance gene in *Hfx. volcanii*. The latter results from a mutation in the chromosomal *hmgA* gene, which can lead to recombination between the chromosome and the resistance gene, resulting in constitutive resistance to this antibiotic. It is therefore important to use a resistance gene from another species, such as the mevinolin resistance gene from *Haloarcula hispanica*.

To avoid this, it is possible to use auxotrophy markers. Numerous markers have already been developed in haloarchaea, in particular in *Hfx. volcanii*. The auxotrophy markers are as follows:

- Uracil auxotrophy: this is based on the *pyrE2* gene of *Hfx. volcanii*, encoding an oroate phosphoribosyl transferase, based on the *pyrF* gene of *Haloferax mediterranei/Har. hispanica* or based on *ura3* of *Hbt. salinarum*, encoding an orotidine-5′-phosphate decarboxylase. This selectable marker allows counter-selection by the addition of 5-fluoro-orotic acid (5-FOA), which inhibits the growth of strains with the wild-type *pyrF* gene. A loss of growth results from the inhibition of nucleic acid synthesis [4,12,91].
- Leucine auxotrophy: this is based on the *leuB* gene of *Hfx. volcanii*, which encodes 3-isopropylmalate dehydrogenase, essential for leucine biosynthesis [32].
- Tryptophan auxotrophy: this is based on the *Hfx. volcanii trpA* gene encoding one of the two subunits of tryptophan synthase [32].
- Thymidine auxotrophy: this is based on the *Hfx. volcanii hdrB* gene encoding a thymidylate synthase [4,12,46].
- Methionine auxotrophy: this is based on the *Hfx. volcanii metX* gene encoding a homoserine O-acetyltransferase [12].
- Histidine auxotrophy: this is based on the *Hfx. volcanii hisC* gene encoding a histidinol-phosphate aminotransferase [12].

Selection markers are currently very limited, both in terms of number and target species. Given their importance, it is essential to increase their diversity and, above all, their targets. This is particularly true for auxotrophy markers, which target only two haloarchaeal species. Minimal media are already available for other haloarchaea, [92] and it is therefore conceivable to develop such markers.

#### 3.3.3. Promoters

Different types of promoters can be used to drive transcription of the target gene. Inducible promoters allow the production of a gene of interest to be controlled by the addition of an inducer. They have no basal activity and can be activated/deactivated by changing the culture conditions. Several types of inducible promoters have been developed for haloarchaea. One of the most widely used inducible promoters is the tryptophanase inducible tryptophan promoter. This promoter, developed in *Hfx. volcanii*, is strongly repressed in the absence of tryptophan and rapidly induced by the addition of ≥1 mM tryptophan [4,12,32,47]. A potassium-inducible promoter is also available. This was constructed from the promoter of the potassium uptake system operon (P*kdp*) of *Hbt. salinarum*, which is progressively inducible to potassium [32]. A heat-inducible promoter has also been established in *Hbt. salinarum*. This promoter is based on the *hsp5* gene, which encodes for a heat shock protein [93].

The current alternative is constitutive and strong promoters, which allow a gene of interest to be continuously overexpressed. Only a few promoters have been developed in halophiles. The P*fdx* promoter (promoter of the gene encoding ferredoxin) is a strong promoter used in *Hfx. volcanii*. Ferredoxin is an important electron transporter in haloarchaea. It is involved in the decarboxylation of α-ketoacids [12]. The 16S RNA promoter is also used in *Hbt. salinarum*. This strong promoter has the advantage of being active throughout growth [94]. Finally, a synthetic promoter has been developed in *Hfx. volcanii*. This promoter is an excellent alternative to the current inducible promoter, as it is able to give yields three times higher than the tryptophan inducible promoter [47,48].

#### 3.3.4. Vectors

Shuttle vectors, suicide vectors and overexpression vectors are available in haloarchaea. To avoid listing them all, only a few examples are given here. For example, the pRo-5 shuttle vector was developed in *Natrialba magadii* and works in several haloarchaea [67], the pUC19 vector work as suicide vector in *Nmn. pharaonis* [79], and thus far, only one overexpression vector is available, the pTA963 vector developed in *Hfx. volcanii*, which is based on a tryptophan inducible promoter [4]. As described above, this type of inducer is not suitable for industrial applications [47]. It is therefore essential to develop new overexpression vectors that are suitable for industrial use.

Vectors are based on a variety of replication origins. They can be derived from native plasmids, such as plasmid pTA230 with the pHV2 origin of replication and plasmid pTA354 with the pHV1/4 origin, both from *Hfx. volcanii* [4,38]. They can also be derived from archaeal viral elements, such as the plasmid pRo-5 from *Na. magadii* based on the *repH* replication origin of the virus ΦCH1 [67]. Finally, vectors can be based on replication origins of plasmids developed for other archaea (such as the plasmid pNB102 developed from a plasmid of *Natronobacterium sp*. AS7091 and which is used in *Na. magadii*) [67].

#### 3.3.5. Gene Knock-Out Methods

The deletion of genes of interest is made possible by knock-out methods. These are knock-out systems that involve the transformation of a suicide vector containing a selectable marker (such as an auxotrophy marker or antibiotic resistance) and using the flanking region of the target to suppress a gene in an auxotrophic or an antibiotic-sensitive strain. Knock-out mutants are selected after screening (single crossing) on a selective medium. It may be worthwhile to favor auxotrophic systems, as they allow cross-selection. Uracil auxotrophy can be combined with cross-selection with 5-FOA. The effectiveness of this system has been tested in haloarchaea such as *Hfx. mediterranei* (*ctrb* genes), *Har. hispanica* (phytoene synthase) or in *Hfx. volcanii*, and in haloalkaliphilic archaea [79,91,95]. The genetic manipulation of haloarchaea is therefore mainly performed using homologous recombination systems. However, it may be interesting to test genome deletion/mutation/editing systems based on CRISPR (clustered regularly interspaced short palindromic repeats)-Cas. Currently, only the CRISPR interference system has been developed in *Hfx. volcanii* [96]. It is crucial that all CRISPR-based methods be implemented in the near future to help work with these new chassis.

## 4. Future Perspectives

As mentioned above, the existence of a robust haloarchaeal chassis is a prerequisite for the production of halophilic extremozymes. Currently, the number/diversity of genetic tools and the lack of optimal strains (for the production of these enzymes) remain the major obstacles to its development. The available tools described in this review provide a solid basis. However, efforts must be made to adapt these tools to industrial overproduction. This applies in particular to the overexpression vectors and deletion strains available.

Currently, only the deletion of the Mrr restriction system gene has been tested in *Hfx. volcanii*. The deletion of *mrr* prevents the cleavage of methylated foreign DNA at the dam and dcm sites, thereby increasing the rate of transformation in *Hfx. volcanii* [4]. Although this deletion is interesting for the construction of a cellular chassis, it is not sufficient. Other cellular activities can interfere with the overproduction of extremozymes. This is particularly true of proteases, which can significantly reduce the number of proteins produced. It is therefore necessary to test the effect of deleting target proteases on the overproduction of extremozymes. Once the proteases that interfere with the production of extremozymes have been identified, a protease-deficient strain can be developed and made available for the production of the extremozymes of interest.

The development of an efficient and cost-effective overexpression vector is also a major issue. Currently, only one inducible overexpression vector is available. This is the tryptophan-inducible overexpression vector from *Hfx. volcanii* [4,47]. Although this vector appears to be effective, it relies on an inducer that can be consumed by the cell. The use of such an inducer is not suitable for industry because it is too expensive. It is therefore necessary to find inducers similar to tryptophan and, above all, to identify alternative inducible promoters that are more effective and less costly.

Without an effective vector and an optimal strain, a haloarchaeal chassis will not be able to compete with conventional mesophilic chassis and the associated costly purification methods. Once these two major issues have been resolved, it will be possible to produce and use halophilic extremozymes in industry. The chassis will have all the necessary characteristics (Figure 2): it will be a haloarchaea (an intracellular medium suitable for the production of halophilic extremozymes), the chassis genome will be available (deletion of genes that could interfere with overproduction), it will have suitable genetic tools (transformation protocol, overexpression vector, knockout system, etc.) and it will have as few proteases and peptidases as possible.

As haloarchaea are often polyextremophilic, it would also be interesting to extend the range of cellular chassis to include such species (e.g., haloalkaliphiles). It would then be possible to propose a chassis capable of producing different types of extremozymes.

Although cellular chassis based on various haloarchaeal species are an excellent alternative, it would also be possible to offer cell-free production. These systems may counteract the deleterious effects of certain heterologous proteins on cell physiology. This would also avoid the generation of deletion strains and the resulting effects on chassis physiology [97]. Such systems have never been described for haloarchaea.

## 5. Conclusions

The transition to greener processes is one of the major challenges facing industry. In this context, extremozymes offer an excellent alternative to traditional chemical catalysts. These enzymes, isolated from extremophilic organisms, are able to maintain optimal activity in extreme industrial environments. However, the lack of a suitable production chassis limits their use. This is particularly true for halophilic enzymes. Due to their adaptation to high salinity, the production of halophilic enzymes requires a haloarchaeal-based chassis, which is not yet available. The development of such a chassis will make it possible to provide functional halophilic enzymes without the need for costly purification steps (unlike mesophilic chassis). In recent years, progress has been made in the genetic manipulation of haloarchaea, but it remains modest. Genetic tools are limited to a few species and are not suitable for industrial applications. Overexpression vectors, fundamental tools for enzyme production, are limited to a single vector based on a tryptophan-inducible promoter. Although effective, this vector requires an inducer that is too expensive for industrial applications. In addition, very few genetic modifications favoring protein overproduction have been tested in haloarchaea. Further efforts are therefore needed to develop new genetic tools, particularly overexpression vectors, and to test genetic modifications to turn haloarchaea into robust chassis for the production of halophilic extremozymes.

## Data Availability

No new data were created.

## Supplementary Materials

The following supporting information can be downloaded at: https://www.mdpi.com/article/10.3390/microorganisms12081738/s1, Table S1: Comparison of haloarchaea according to the characteristics required for a cellular chassis.

## References

[1] Chapman J., Ismail A., Dinu C. (2018). Industrial Applications of Enzymes: Recent Advances, Techniques, and Outlooks. Catalysts.

[2] Espina G., Muñoz-Ibacache S.A., Cáceres-Moreno P., Amenabar M.J., Blamey J.M. (2022). From the Discovery of Extremozymes to an Enzymatic Product: Roadmap Based on Their Applications. Front. Bioeng. Biotechnol..

[3] Delgado-García M., Valdivia-Urdiales B., Aguilar-González C.N., Contreras-Esquivel J.C., Rodríguez-Herrera R. (2012). Halophilic Hydrolases as a New Tool for the Biotechnological Industries. J. Sci. Food Agric..

[4] Allers T., Barak S., Liddell S., Wardell K., Mevarech M. (2010). Improved Strains and Plasmid Vectors for Conditional Overexpression of His-Tagged Proteins in *Haloferax volcanii*. Appl. Environ. Microbiol..

[5] Munawar N., Engel P.C. (2013). Halophilic Enzymes: Characteristics, Structural Adaptation and Potential Applications for Biocatalysis. Curr. Biotechnol..

[6] Matarredona L., Camacho M., Zafrilla B., Bonete M.-J., Esclapez J. (2020). The Role of Stress Proteins in Haloarchaea and Their Adaptive Response to Environmental Shifts. Biomolecules.

[7] Moopantakath J., Imchen M., Anju V.T., Busi S., Dyavaiah M., Martínez-Espinosa R.M., Kumavath R. (2023). Bioactive Molecules from Haloarchaea: Scope and Prospects for Industrial and Therapeutic Applications. Front. Microbiol..

[8] Wood R.B., Talling J.F. (1988). Chemical and Algal Relationships in a Salinity Series of Ethiopian Inland Waters. Hydrobiologia.

[9] Oren A., Dworkin M., Falkow S., Rosenberg E., Schleifer K.-H., Stackebrandt E. (2006). Life at High Salt Concentrations. The Prokaryotes.

[10] Becker E.A., Seitzer P.M., Tritt A., Larsen D., Krusor M., Yao A.I., Wu D., Madern D., Eisen J.A., Darling A.E. (2014). Phylogenetically Driven Sequencing of Extremely Halophilic Archaea Reveals Strategies for Static and Dynamic Osmo-Response. PLoS Genet..

[11] Thombre R.S., Shinde V.D., Oke R.S., Dhar S.K., Shouche Y.S. (2016). Biology and Survival of Extremely Halophilic Archaeon *Haloarcula marismortui* RR12 Isolated from Mumbai Salterns, India in Response to Salinity Stress. Sci. Rep..

[12] Leigh J.A., Albers S.-V., Atomi H., Allers T. (2011). Model Organisms for Genetics in the Domain Archaea: Methanogens, Halophiles, Thermococcales and Sulfolobales. FEMS Microbiol. Rev..

[13] Hermann L., Mais C.-N., Czech L., Smits S.H.J., Bange G., Bremer E. (2020). The Ups and Downs of Ectoine: Structural Enzymology of a Major Microbial Stress Protectant and Versatile Nutrient. Biol. Chem..

[14] Orellana R., Macaya C., Bravo G., Dorochesi F., Cumsille A., Valencia R., Rojas C., Seeger M. (2018). Living at the Frontiers of Life: Extremophiles in Chile and Their Potential for Bioremediation. Front. Microbiol..

[15] Kokoeva M.V. (2002). A Novel Mode of Sensory Transduction in Archaea: Binding Protein-Mediated Chemotaxis towards Osmoprotectants and Amino Acids. EMBO J..

[16] Youssef N.H., Savage-Ashlock K.N., McCully A.L., Luedtke B., Shaw E.I., Hoff W.D., Elshahed M.S. (2014). Trehalose/2-Sulfotrehalose Biosynthesis and Glycine-Betaine Uptake Are Widely Spread Mechanisms for Osmoadaptation in the *Halobacteriales*. ISME J..

[17] Litchfield C.D. (2011). Potential for Industrial Products from the Halophilic *Archaea*. J. Ind. Microbiol. Biotechnol..

[18] DasSarma P., Zamora R.C., Müller J.A., DasSarma S. (2012). Genome-Wide Responses of the Model Archaeon *Halobacterium* Sp. Strain NRC-1 to Oxygen Limitation. J. Bacteriol..

[19] Weerasooriya R.R., Liyanage L.P.K., Rathnappriya R.H.K., Bandara W.B.M.A.C., Perera T.A.N.T., Gunarathna M.H.J.P., Jayasinghe G.Y. (2021). Industrial Water Conservation by Water Footprint and Sustainable Development Goals: A Review. Environ. Dev. Sustain..

[20] Cho E.-S., Cha I.-T., Roh S.W., Seo M.-J. (2021). *Haloferax litoreum* Sp. Nov., *Haloferax marinisediminis* Sp. Nov., and *Haloferax marinum* Sp. Nov., Low Salt-Tolerant Haloarchaea Isolated from Seawater and Sediment. Antonie van Leeuwenhoek.

[21] Zhang X., Lin Y., Chen G.-Q. (2018). Halophiles as Chassis for Bioproduction. Adv. Biosyst..

[22] Díaz S., Pérez-Pomares F., Pire C., Ferrer J., Bonete M.-J. (2006). Gene Cloning, Heterologous Overexpression and Optimized Refolding of the NAD-Glutamate Dehydrogenase from Haloferax Mediterranei. Extremophiles.

[23] Calero P., Nikel P.I. (2019). Chasing Bacterial Chassis for Metabolic Engineering: A Perspective Review from Classical to Non-Traditional Microorganisms. Microb. Biotechnol..

[24] Kim K., Choe D., Lee D.-H., Cho B.-K. (2020). Engineering Biology to Construct Microbial Chassis for the Production of Difficult-to-Express Proteins. Int. J. Mol. Sci..

[25] Oren A., Rosenberg E., DeLong E.F., Lory S., Stackebrandt E., Thompson F. (2014). The Family *Halobacteriaceae*. The Prokaryotes.

[26] National Center for Biotechnology Information (NCBI). https://www.ncbi.nlm.nih.gov/.

[27] Roh S.W., Nam Y.-D., Chang H.-W., Sung Y., Kim K.-H., Oh H.-M., Bae J.-W. (2007). *Halalkalicoccus jeotgali* Sp. Nov., a Halophilic Archaeon from Shrimp Jeotgal, a Traditional Korean Fermented Seafood. Int. J. Syst. Evol. Microbiol..

[28] Xue Y., Fan H., Ventosa A., Grant W.D., Jones B.E., Cowan D.A., Ma Y. (2005). *Halalkalicoccus tibetensis* Gen. Nov., Sp. Nov., Representing a Novel Genus of Haloalkaliphilic Archaea. Int. J. Syst. Evol. Microbiol..

[29] Liu B.-B., Narsing Rao M.P., Yin X.-Q., Li X., Salam N., Zhang Y., Alkhalifah D.H.M., Hozzein W.N., Li W.-J. (2019). Description of *Halegenticoccus soli* Gen. Nov., Sp. Nov., a Halophilic Archaeon Isolated from a Soil Sample of Ebi Lake. Extremophiles.

[30] Liu B.-B., Salam N., Cheng S., Zhang W., Zhou Y., Guo S., Li W.-J. (2021). *Halegenticoccus tardaugens* Sp. Nov., an Extremely Halophilic Archaeon Isolated from a Saline Soil. Extremophiles.

[31] Cline S.W., Doolittle W.F. (1992). Transformation of Members of the Genus Haloarcula with Shuttle Vectors Based on *Halobacterium halobium* and *Haloferax volcanii* Plasmid Replicons. J. Bacteriol..

[32] Atomi H., Imanaka T., Fukui T. (2012). Overview of the Genetic Tools in the Archaea. Front. Microbiol..

[33] Juez G., Rodriguez-Valera F., Ventosa A., Kushner D.J. (1986). *Haloarcula hispanica* Spec. Nov. and *Haloferax gibbonsii* Spec, Nov., Two New Species of Extremely Halophilic Archaebacteria. Syst. Appl. Microbiol..

[34] Nakamura S., Nakasone K., Takashina T., Horikoshi K. (2011). Genetics and Genomics of Triangular Disc-Shaped Halophilic Archaeon *Haloarcula japonica* Strain TR-1. Extremophiles Handbook.

[35] Baliga N.S., Bonneau R., Facciotti M.T., Pan M., Glusman G., Deutsch E.W., Shannon P., Chiu Y., Weng R.S., Gan R.R. (2004). Genome Sequence of *Haloarcula marismortui*: A Halophilic Archaeon from the Dead Sea. Genome Res..

[36] Kixmüller D., Greie J.-C. (2012). Construction and Characterization of a Gradually Inducible Expression Vector for *Halobacterium Salinarum*, Based on the Kdp Promoter. Appl. Environ. Microbiol..

[37] Haloarchaea. https://haloarchaea.com/halohandbook/.

[38] Patenge N., Haase A., Bolhuis H., Oesterhelt D. (2000). The Gene for a Halophilic β-Galactosidase (bgaH) of *Haloferax alicantei* as a Reporter Gene for Promoter Analyses in *Halobacterium Salinarum*. Mol. Microbiol..

[39] Shimoshige H., Yamada T., Minegishi H., Echigo A., Shimane Y., Kamekura M., Itoh T., Usami R. (2013). *Halobaculum magnesiiphilum* Sp. Nov., a Magnesium-Dependent Haloarchaeon Isolated from Commercial Salt. Int. J. Syst. Evol. Microbiol..

[40] Minegishi H., Echigo A., Kuwahara A., Shimane Y., Kamekura M., Itoh T., Ohkuma M., Usami R. (2015). *Halocalculus aciditolerans* Gen. Nov., Sp. Nov., an Acid-Tolerant Haloarchaeon Isolated from Commercial Salt. Int. J. Syst. Evol. Microbiol..

[41] Verma A., Pal Y., Kumar P., Krishnamurthi S. (2020). *Halocatena pleomorpha* Gen. Nov. Sp. Nov., an Extremely Halophilic Archaeon of Family *Halobacteriaceae* Isolated from Saltpan Soil. Int. J. Syst. Evol. Microbiol..

[42] Sorokin D.Y., Messina E., Smedile F., Roman P., Damsté J.S.S., Ciordia S., Mena M.C., Ferrer M., Golyshin P.N., Kublanov I.V. (2017). Discovery of Anaerobic Lithoheterotrophic Haloarchaea, Ubiquitous in Hypersaline Habitats. ISME J..

[43] Xu X.-W., Wu Y.-H., Wang C.-S., Oren A., Zhou P.-J., Wu M. (2007). *Haloferax larsenii* Sp. Nov., an Extremely Halophilic Archaeon from a Solar Saltern. Int. J. Syst. Evol. Microbiol..

[44] Haque R.U., Paradisi F., Allers T. (2020). *Haloferax volcanii* for Biotechnology Applications: Challenges, Current State and Perspectives. Appl. Microbiol. Biot..

[45] Cline S.W., Schalkwyk L.C., Doolittle W.F. (1989). Transformation of the Archaebacterium Halobacterium Volcanii with Genomic DNA. J. Bacteriol..

[46] Ortenberg R., Rozenblatt-Rosen O., Mevarech M. (2002). The Extremely Halophilic Archaeon *Haloferax volcanii* Has Two Very Different Dihydrofolate Reductases: *Haloferax volcanii* Dihydrofolate Reductases. Mol. Microbiol..

[47] Large A., Stamme C., Lange C., Duan Z., Allers T., Soppa J., Lund P.A. (2007). Characterization of a Tightly Controlled Promoter of the Halophilic Archaeon *Haloferax volcanii* and Its Use in the Analysis of the Essential Cct1 Gene. Mol. Microbiol..

[48] Haque R.U., Paradisi F., Allers T. (2019). *Haloferax volcanii* as Immobilised Whole Cell Biocatalyst: New Applications for Halophilic Systems. Appl. Microbiol. Biotechnol..

[49] Lam W.L., Doolittle W.F. (1989). Shuttle Vectors for the Archaebacterium *Halobacterium volcanii*. Proc. Natl. Acad. Sci. USA.

[50] Huber M., Soppa J. (2019). Dihydrofolate Reductase (DHFR) Reporter Enzyme Assay for Haloferax volcanii.

[51] Reuter C.J., Maupin-Furlow J.A. (2004). Analysis of Proteasome-Dependent Proteolysis in *Haloferax volcanii* Cells, Using Short-Lived Green Fluorescent Proteins. Appl. Environ. Microbiol..

[52] Savage K.N., Krumholz L.R., Oren A., Elshahed M.S. (2008). *Halosarcina pallida* Gen. Nov., Sp. Nov., a Halophilic Archaeon from a Low-Salt, Sulfide-Rich Spring. Int. J. Syst. Evol. Microbiol..

[53] Durán-Viseras A., Sánchez-Porro C., Ventosa A. (2020). *Haloglomus irregulare* Gen. Nov., Sp. Nov., a New Halophilic Archaeon Isolated from a Marine Saltern. Microorganisms.

[54] Mou Y.-Z., Qiu X.-X., Zhao M.-L., Cui H.-L., Oh D., Dyall-Smith M.L. (2012). *Halohasta litorea* Gen. Nov. Sp. Nov., and *Halohasta litchfieldiae* Sp. Nov., Isolated from the Daliang Aquaculture Farm, China and from Deep Lake, Antarctica, Respectively. Extremophiles.

[55] Inoue K., Itoh T., Ohkuma M., Kogure K. (2011). *Halomarina oriensis* Gen. Nov., Sp. Nov., a Halophilic Archaeon Isolated from a Seawater Aquarium. Int. J. Syst. Evol. Microbiol..

[56] Echigo A., Minegishi H., Shimane Y., Kamekura M., Itoh T., Usami R. (2013). *Halomicroarcula pellucida* Gen. Nov., Sp. Nov., a Non-Pigmented, Transparent-Colony-Forming, Halophilic Archaeon Isolated from Solar Salt. Int. J. Syst. Evol. Microbiol..

[57] Chen S., Xu Y., Sun S., Liu J., Chen F. (2020). *Halomicrococcus hydrotolerans* Gen. Nov., Sp. Nov., an Extremely Halophilic Archaeon Isolated from a Subterranean Salt Deposit. Int. J. Syst. Evol. Microbiol..

[58] Kondo Y., Minegishi H., Echigo A., Shimane Y., Kamekura M., Itoh T., Ohkuma M., Tanaka A., Takahashi-Ando N., Fukushima Y. (2016). *Haloparvum alkalitolerans* Sp. Nov., Alkali-Tolerant Haloarchaeon Isolated from Commercial Salt. Int. J. Syst. Evol. Microbiol..

[59] Chen S., Liu H.-C., Zhou J., Xiang H. (2016). *Haloparvum sedimenti* Gen. Nov., Sp. Nov., a Member of the Family Haloferacaceae. Int. J. Syst. Evol. Microbiol..

[60] Song H.S., Cha I.-T., Yim K.J., Lee H.-W., Hyun D.-W., Lee S.-J., Rhee S.-K., Kim K.-N., Kim D., Choi J.-S. (2014). *Halapricum Salinum* Gen. Nov., Sp. Nov., an Extremely Halophilic Archaeon Isolated from Non-Purified Solar Salt. Antonie van Leeuwenhoek J. Microb..

[61] Xu Q., Cui H.-L., Meng F. (2019). *Haloprofundus halophilus* Sp. Nov., Isolated from the Saline Soil of Tarim Basin. Antonie van Leeuwenhoek.

[62] Zhang G., Gu J., Zhang R., Rashid M., Haroon M.F., Xun W., Ruan Z., Dong X., Stingl U. (2017). *Haloprofundus marisrubri* Gen. Nov., Sp. Nov., an Extremely Halophilic Archaeon Isolated from a Brine–Seawater Interface. Int. J. Syst. Evol. Microbiol..

[63] Haloweb. https://haloweb.org/.

[64] Antunes A., Taborda M., Huber R., Moissl C., Nobre M.F., da Costa M.S. (2008). *Halorhabdus tiamatea* Sp. Nov., a Non-Pigmented, Extremely Halophilic Archaeon from a Deep-Sea, Hypersaline Anoxic Basin of the Red Sea, and Emended Description of the Genus *Halorhabdus*. Int. J. Syst. Evol. Microbiol..

[65] Wainø M., Tindall B.J., Ingvorsen K. (2000). *Halorhabdus utahensis* Gen. Nov., Sp. Nov., an Aerobic, Extremely Halophilic Member of the Archaea from Great Salt Lake, Utah. Int. J. Syst. Evol. Microbiol..

[66] Cui H.-L., Mou Y.-Z., Yang X., Zhou Y.-G., Liu H.-C., Zhou P.-J. (2012). *Halorubellus salinus* Gen. Nov., Sp. Nov. and *Halorubellus litoreus* Sp. Nov., Novel Halophilic Archaea Isolated from a Marine Solar Saltern. Syst. Appl. Microbiol..

[67] Mayrhofer-Iro M., Ladurner A., Meissner C., Derntl C., Reiter M., Haider F., Dimmel K., Rössler N., Klein R., Baranyi U. (2013). Utilization of Virus ϕCh1 Elements To Establish a Shuttle Vector System for Halo(Alkali)Philic Archaea via Transformation of *Natrialba magadii*. Appl. Environ. Microbiol..

[68] Liao Y., Williams T.J., Walsh J.C., Ji M., Poljak A., Curmi P.M.G., Duggin I.G., Cavicchioli R. (2016). Developing a Genetic Manipulation System for the Antarctic Archaeon, *Halorubrum Lacusprofundi*: Investigating Acetamidase Gene Function. Sci. Rep..

[69] Cui H.-L., Gao X., Yang X., Xu X.-W. (2010). *Halorussus rarus* Gen. Nov., Sp. Nov., a New Member of the Family Halobacteriaceae Isolated from a Marine Solar Saltern. Extremophiles.

[70] Mehrshad M., Amoozegar M.A., Makhdoumi A., Fazeli S.A.S., Farahani H., Asadi B., Schumann P., Ventosa A. (2016). *Halosiccatus urmianus* Gen. Nov., Sp. Nov., a Haloarchaeon from a Salt Lake. Int. J. Syst. Evol. Microbiol..

[71] Han D., Hong L.-G., Xu Q., Cui H.-L. (2019). *Halostella limicola* Sp. Nov., Isolated from Saline Soil Sampled at the Tarim Basin. Int. J. Syst. Evol. Microbiol..

[72] Han D., Cui H.-L. (2020). *Halostella pelagica* Sp. Nov. and *Halostella litorea* Sp. Nov., Isolated from Salted Brown Alga *Laminaria*. Int. J. Syst. Evol. Microbiol..

[73] Song H.S., Cha I.-T., Rhee J.-K., Yim K.J., Kim A.Y., Choi J.-S., Baek S.J., Seo M.-J., Park S.-J., Nam Y.-D. (2016). *Halostella salina* Gen. Nov., Sp. Nov., an Extremely Halophilic Archaeon Isolated from Solar Salt. Int. J. Syst. Evol. Microbiol..

[74] Mehrshad M., Amoozegar M.A., Makhdoumi A., Rasooli M., Asadi B., Schumann P., Ventosa A. (2015). *Halovarius luteus* Gen. Nov., Sp. Nov., an Extremely Halophilic Archaeon from a Salt Lake. Int. J. Syst. Evol. Microbiol..

[75] Liu Q., Ren M., Zhang L.-L. (2015). *Natribaculum breve* Gen. Nov., Sp. Nov. and *Natribaculum longum* Sp. Nov., Halophilic Archaea Isolated from Saline Soil. Int. J. Syst. Evol. Microbiol..

[76] Romano I., Poli A., Finore I., Huertas F.J., Gambacorta A., Pelliccione S., Nicolaus G., Lama L., Nicolaus B. (2007). *Haloterrigena hispanica* Sp. Nov., an Extremely Halophilic Archaeon from Fuente de Piedra, Southern Spain. Int. J. Syst. Evol. Microbiol..

[77] Sorokin D.Y., Khijniak T.V., Kostrikina N.A., Elcheninov A.G., Toshchakov S.V., Bale N.J., Damsté J.S.S., Kublanov I.V. (2018). *Natronobiforma cellulositropha* Gen. Nov., Sp. Nov., a Novel Haloalkaliphilic Member of the Family Natrialbaceae (Class Halobacteria) from Hypersaline Alkaline Lakes. Syst. Appl. Microbiol..

[78] Zhao B., Hu Q., Guo X., Liao Z., Sarmiento F., Mesbah N.M., Yan Y., Li J., Wiegel J. (2018). *Natronolimnobius aegyptiacus* Sp. Nov., an Extremely Halophilic Alkalithermophilic Archaeon Isolated from the Athalassohaline Wadi An Natrun, Egypt. Int. J. Syst. Evol. Microbiol..

[79] Orsini S.S., James K.L., Reyes D.J., Couto-Rodriguez R.L., Gulko M.K., Witte A., Carroll R.K., Rice K.C. (2020). Bacterial-like Nitric Oxide Synthase in the Haloalkaliphilic Archaeon *Natronomonas pharaonis*. MicrobiologyOpen.

[80] Cui H.-L., Tohty D., Liu H.-C., Liu S.-J., Oren A., Zhou P.-J. (2007). *Natronorubrum sulfidifaciens* Sp. Nov., an Extremely Haloalkaliphilic Archaeon Isolated from Aiding Salt Lake in Xin-Jiang, China. Int. J. Syst. Evol. Microbiol..

[81] Ruiz-Romero E., Valenzuela-Encinas C., López-Ramírez M.P., de los Angeles Coutiño-Coutiño M., Marsch R., Dendooven L. (2013). *Natronorubrum texcoconense* Sp. Nov., a Haloalkaliphilic Archaeon Isolated from Soil of the Former Lake Texcoco (Mexico). Arch. Microbiol..

[82] Cui H.-L., Yang X., Mou Y.-Z. (2011). *Salinarchaeum laminariae* Gen. Nov., Sp. Nov.: A New Member of the Family Halobacteriaceae Isolated from Salted Brown Alga Laminaria. Extremophiles.

[83] Han D., Cui H.-L. (2020). *Salinibaculum litoreum* Gen. Nov., Sp. Nov., Isolated from Salted Brown Alga Laminaria. Int. J. Syst. Evol. Microbiol..

[84] Cui H.-L., Zhang W.-J. (2014). *Salinigranum rubrum* Gen. Nov., Sp. Nov., a Member of the Family Halobacteriaceae Isolated from a Marine Solar Saltern. Int. J. Syst. Evol. Microbiol..

[85] Wang Z., Xu J.-Q., Xu W.-M., Li Y., Zhou Y., Lü Z.-Z., Hou J., Zhu L., Cui H.-L. (2016). *Salinigranum salinum* Sp. Nov., Isolated from a Marine Solar Saltern. Int. J. Syst. Evol. Microbiol..

[86] Hou J., Zhao Y.-J., Zhu L., Cui H.-L. (2018). *Salinirubellus salinus* Gen. Nov., Sp. Nov., Isolated from a Marine Solar Saltern. Int. J. Syst. Evol. Microbiol..

[87] Cui H.-L., Qiu X.-X. (2014). *Salinarubrum litoreum* Gen. Nov., Sp. Nov.: A New Member of the Family Halobacteriaceae Isolated from Chinese Marine Solar Salterns. Antonie van Leeuwenhoek.

[88] Cui H.-L., Lü Z.-Z., Li Y., Zhou Y. (2017). *Salinirussus salinus* Gen. Nov., Sp. Nov., Isolated from a Marine Solar Saltern. Int. J. Syst. Evol. Microbiol..

[89] Yin X.-Q., Liu B.-B., Chu X., Salam N., Li X., Yang Z.-W., Zhang Y., Xiao M., Li W.-J. (2017). *Saliphagus infecundisoli* Gen. Nov., Sp. Nov., an Extremely Halophilic Archaeon Isolated from a Saline Soil. Int. J. Syst. Evol. Microbiol..

[90] Cline S.W., Lam W.L., Charlebois R.L., Schalkwyk L.C., Doolittle W.F. (1989). Transformation Methods for Halophilic Archaebacteria. Can. J. Microbiol..

[91] Liu H., Han J., Liu X., Zhou J., Xiang H. (2011). Development of pyrF-Based Gene Knockout Systems for Genome-Wide Manipulation of the Archaea *Haloferax mediterranei* and *Haloarcula hispanica*. J. Genet. Genom..

[92] Falb M., Pfeiffer F., Palm P., Rodewald K., Hickmann V., Tittor J., Oesterhelt D. (2005). Living with Two Extremes: Conclusions from the Genome Sequence of *Natronomonas pharaonis*. Genome Res..

[93] Lu Q., Han J., Zhou L., Coker J.A., DasSarma P., DasSarma S., Xiang H. (2008). Dissection of the Regulatory Mechanism of a Heat-Shock Responsive Promoter in Haloarchaea: A New Paradigm for General Transcription Factor Directed Archaeal Gene Regulation. Nucleic Acids Res..

[94] Born J., Pfeifer F. (2019). Improved GFP Variants to Study Gene Expression in Haloarchaea. Front. Microbiol..

[95] Derntl C., Selb R., Klein R., Alte B., Witte A. (2015). Genomic Manipulations in Alkaliphilic Haloarchaea Demonstrated by a Gene Disruption in *Natrialba magadii*. FEMS Microbiol. Lett..

[96] Gophna U., Allers T., Marchfelder A. (2017). Finally, Archaea Get Their CRISPR-Cas Toolbox. Trends Microbiol..

[97] Khambhati K., Bhattacharjee G., Gohil N., Braddick D., Kulkarni V., Singh V. (2019). Exploring the Potential of Cell-Free Protein Synthesis for Extending the Abilities of Biological Systems. Front. Bioeng. Biotechnol..

